# Peritoneal Tumorigenesis and Inflammation are Ameliorated by Humidified-Warm Carbon Dioxide Insufflation in the Mouse

**DOI:** 10.1245/s10434-015-4508-1

**Published:** 2015-03-21

**Authors:** Sandra Carpinteri, Shienny Sampurno, Maria-Pia Bernardi, Markus Germann, Jordane Malaterre, Alexander Heriot, Brenton A. Chambers, Steven E. Mutsaers, Andrew C. Lynch, Robert G. Ramsay

**Affiliations:** 1 Cancer Research Division, Peter MacCallum Cancer Centre, Melbourne, VIC Australia; 2 Department of Surgical Oncology, Peter MacCallum Cancer Centre, Melbourne, VIC Australia; 3 Faculty of Veterinary Science, University of Melbourne, Parkville, VIC Australia; 4 Lung Institute of Western Australia and Centre for Cell Therapy and Regenerative Medicine, Nedlands, WA Australia; 5 Peter MacCallum Cancer Centre, Sir Peter MacCallum Department of Oncology, The University of Melbourne, Melbourne, VIC Australia

## Abstract

**Background:**

Conventional laparoscopic surgery uses CO_2_ that is dry and cold, which can damage peritoneal surfaces. It is speculated that disseminated cancer cells may adhere to such damaged peritoneum and metastasize. We hypothesized that insufflation using humidified-warm CO_2_, which has been shown to reduce mesothelial damage, will also ameliorate peritoneal inflammation and tumor cell implantation compared to conventional dry-cold CO_2_.

**Methods:**

Laparoscopic insufflation was modeled in mice along with anesthesia and ventilation. Entry and exit ports were introduced to maintain insufflation using dry-cold or humidified-warm CO_2_ with a constant flow and pressure for 1 h; then 1000 or 1 million fluorescent-tagged murine colorectal cancer cells (CT26) were delivered into the peritoneal cavity. The peritoneum was collected at intervals up to 10 days after the procedure to measure inflammation, mesothelial damage, and tumor burden using fluorescent detection, immunohistochemistry, and scanning electron microscopy.

**Results:**

Rapid temperature control was achieved only in the humidified-warm group. Port-site tumors were present in all mice. At 10 days, significantly fewer tumors on the peritoneum were counted in mice insufflated with humidified-warm compared to dry-cold CO_2_ (*p* < 0.03). The inflammatory marker COX-2 was significantly increased in the dry-cold compared to the humidified-warm cohort (*p* < 0.01), while VEGFA expression was suppressed only in the humidified-warm cohort. Significantly less mesothelial damage and tumor cell implantation was evident from 2 h after the procedure in the humidified-warm cohort.

**Conclusions:**

Mesothelial cell damage and inflammation are reduced by using humidified-warm CO_2_ for laparoscopic oncologic surgery and may translate to reduce patients’ risk of developing peritoneal metastasis.

**Electronic supplementary material:**

The online version of this article (doi:10.1245/s10434-015-4508-1) contains supplementary material, which is available to authorized users.

Laparoscopy is the preferred minimally invasive approach used in a range of abdominal procedures reported to decrease the volume of intraoperative bleeding, reduce the risk of infections and shorten postoperative hospital stays.^1^ Laparoscopy uses CO_2_ gas that is dry and cold to insufflate the abdomen. This may have negative impact on the patient in the short and longer term, as it has been found to increase hypothermia and to exacerbate intra-abdominal adhesions and damage of the mesothelial lining of the peritoneum.^2^,^3^

Up to 35 % of patients die of peritoneal tumor recurrence after curative colorectal cancer resection, often resulting in locoregional morbidity without systemic metastasis.^4^,^5^ Laparoscopic surgery has generated controversy, as it has been argued that disseminated tumor cells may arise during the procedure and may have an increased propensity to metastasize to the peritoneum.^6^,^7^ The underlying basis of this is thought to be due to tissue trauma caused by desiccation, predisposing to tumor cell adhesion potentiated by exposure to dry-cold CO_2_ during laparoscopy. This leads to the proposal that these events might be reduced by using humidified-warm CO_2_ gas for abdominal insufflation. Testing these concepts in mice is effective because large cohort sizes can be used using low cell numbers to generate a minimal cancer burden to reflect the tumor recurrence rates seen in patients. A traceable colorectal cancer model, combined with a shorter time to generate metastasis data after laparoscopy, provide tools to directly test the effects of different insufflation modalities on peritoneal carcinomatosis.

The parietal peritoneum is composed of a single mesothelial layer that covers connective tissue and regulates angiogenesis, fibrinolysis, inflammation, and tissue repair.^8^,^9^ Microvilli projections from a monolayer of mesothelial cells provide a frictionless surface between the peritoneum and visceral lining of the abdominal organs, allowing transport of nutrients and growth factors across the peritoneum.^10^ For sustained hydration, tissue remodeling and regulating inflammation, a thin fluid film, the glycocalyx, overlays the mesothelium.^8^

The first sign of peritoneal damage after injury is a change in mesothelial microvilli, with shortening and progressive disappearance evident during peritoneal dialysis and peritonitis.^10^,^11^ Conversely, during peritoneal recovery, microvilli are more abundant and may ameliorate further damage of the mesothelium, promoting repair by maintaining protection by the glycocalyx.^12^,^13^ The second sign of damage is a change in mesothelial cell morphology, characterized by rounding up of the cells and detachment from the basal lamina.^12^ Events are potentiated by desiccation due to dry-cold CO_2_ exposure.^14^ This combined injury may facilitate mesothelial breach, cancer cell adherence, and implantation on the basement membrane.^15^

Laparoscopy minimizes peritoneal desiccation compared to laparotomy.^16^ However, the use of dry-cold CO_2_ for insufflation may itself modify the mesothelium and promote metastasis.^6^ Peritoneal trauma during surgery elicits the release of proinflammatory and proangiogenic mediators such as cyclooxygenase-2 (COX-2) and vascular endothelial growth factor A (VEGFA) to facilitate wound repair. COX-2 is an enzyme that synthesizes prostaglandins as part of the normal inflammatory response. It also induces angiogenesis after tissue injury.^17^ An increase in VEGFA similarly stimulates angiogenesis after tissue damage to promote reoxygenation for repair.^18^ Increases in these factors may also create a protumorigenic environment, enhancing adhesion of disseminated cancer cells, resulting in peritoneal metastasis and are poor prognosis markers in cancer.^19^,^20^

The aim of the study was to explore the impact of humidification and warming of CO_2_ used for laparoscopic insufflation on peritoneal damage, inflammation and the potential to develop peritoneal metastases in a colorectal mouse model.

## Methods

### Animals and Husbandry

The study was performed in accordance with the animal ethics guidelines of the National Health and Medical Research Council (NHMRC) (Australia) and approved by the Peter MacCallum Cancer Centre (PMCC) Animal Ethics Committee. Mice were purchased from the ARC (Perth, Australia) and housed in the PMCC facility with 12 h light cycles and access to food and water ad libitum. Female BALB/c mice were 9–10 weeks old and weighed 20–22 g.

### Tumor Cells and Retroviral Transduction

Murine stem cell virus (MSCV)-mCherry-CT26 cells were obtained by stable retroviral transduction with an MSCV-mCherry vector derived by replacing the green fluorescent protein (GFP) coding region of MSCV-IRES-GFP (Addgene) with the mCherry coding sequence from pmCherry (Clonetech) [21].

### Insufflation Procedures and Experimental Design

**Fig. 1:**
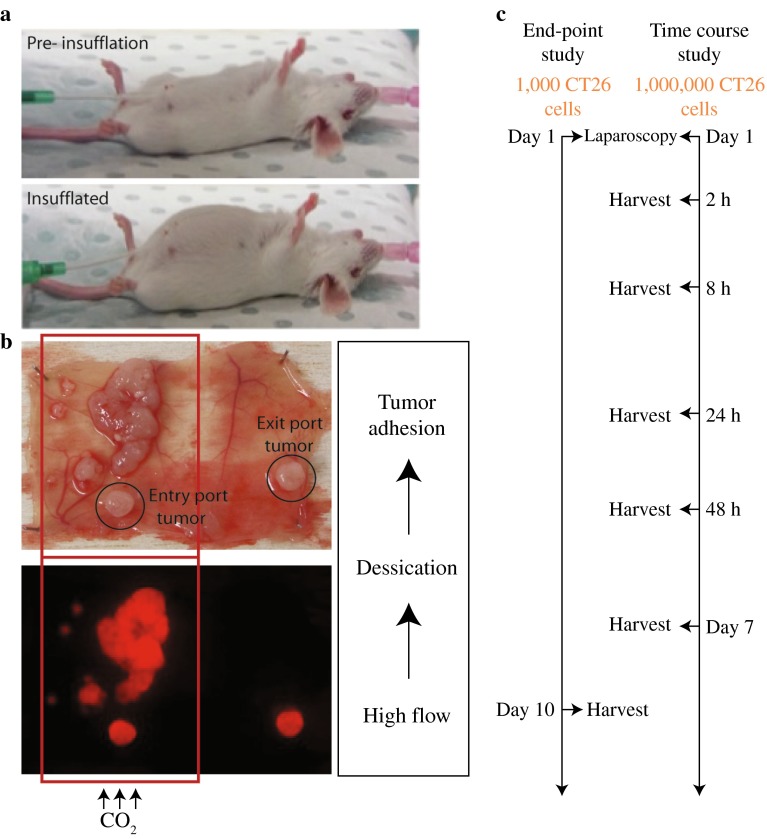
Mouse model of human laparoscopic insufflation. **a** Images of preinsufflated and actively insufflated mice. **b** Tumor detection on peritoneum and at port sites. In pilot trials, we established that elevated gas flow rates, particularly using dry-cold CO_2_, led to excessive and localized tumor development. **c** Work flow design of end point and time course experiments after insufflation designed to allow quantitation of tumor burden and peritoneal inflammatory responses, respectively

The details used to model the consequences of potential tumor cell mobilization and engraftment after laparoscopic insufflation depicted in Fig. 1, and conditions are described in the Supplementary Information. In brief, mice were anesthetized and ventilated before port sites were introduced for insufflation with dry-cold or humidified-warm CO_2_ (Supplementary Fig. 1). Tumor cells were inoculated after 1 h.

### Tissue Collection, Preparation, Immunohistochemistry (IHC), and Scanning Electron Microscopy (SEM)

Tissue collection and processing plus IHC details are described in detail in the Supplementary Information. In brief, sections were stained for COX-2, VEGFA, and F4/80 expression detected and visualized with horseradish peroxidase secondary antibodies. Tissue specimens were examined using a benchtop scanning electron microscope (JCM-6000; Jeol) and operated at 15 kV under high vacuum.

### Fluorescent Image Analysis

Tumors on the peritoneum were analyzed for Cherry-Red fluorescence using the Maestro imaging apparatus (Maestro 2, Cri; Perkin Elmer) with an exposure of 150 nm. The average signal of pixels per tumor area was quantitated by the Maestro software package.

### Semiquantitative Analyses

Mice were culled at appropriate time points and the number of macroscopic tumors counted in a blinded fashion by two individual researchers; their scores were averaged per sample. Exit port tumors were excluded from analysis. Immunolabeling was evaluated in a blinded fashion by two independent investigators using an H score (range 0–12) by scoring intensity (0–3) multiplied by extent (0–4). SEM was used to evaluate changes to morphology and alterations quantified using a customary scale adapted from the H-score method and represented as a percentage.

### Statistical Analyses

All data are expressed as mean ± standard error of the mean. Data were evaluated using GraphPad Prism 6 and analyzed by 1- or 2-way ANOVA with Tukey’s multiple comparisons test or one-tailed unpaired *t* test. A *p* value of less than 0.05 was considered statistically significant.

## Results

### Maintaining Normothermia During Insufflation

**Fig. 2:**
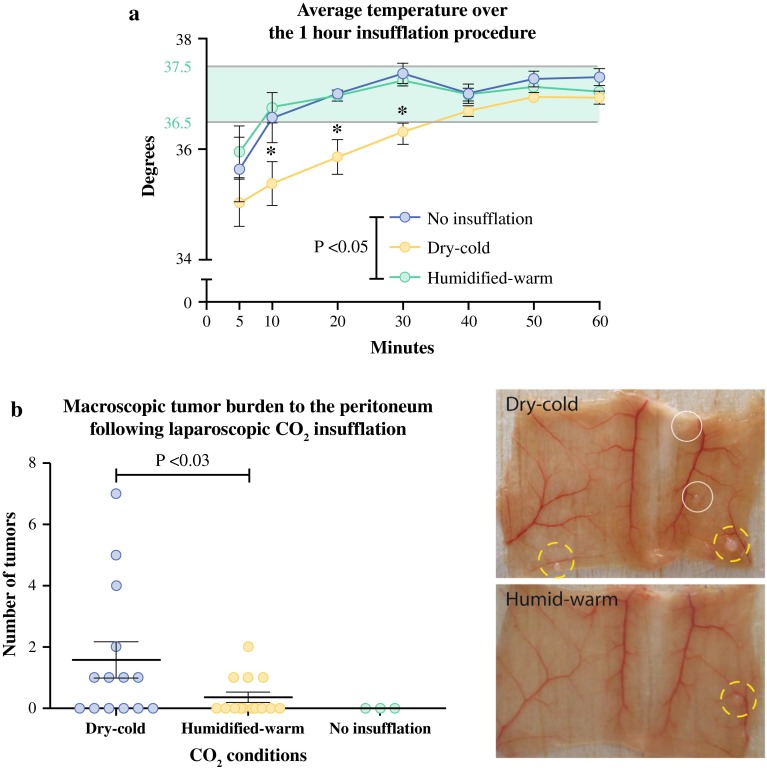
Temperature regulation and tumor burden depending on CO_2_ insufflation protocol. **a** Heat lamp intervention was used when maintaining normothermia within the range 36.5–37.5 °C (*green zone*) throughout procedure for no insufflation control (*n* = 3), dry-cold CO.2 (*n* = 14), and humidified-warm CO_2_ (*n* = 14) cohorts. Significant hypothermia was evident in the dry-cold cohort. **b** Tumor masses were identified macroscopically (*white circles*) on the peritoneum of mice 10 days after insufflation with either dry-cold CO_2_ (*n* = 14) or humidified-warm CO_2_ (*n* = 14), or in mice that were not insufflated (*n* = 3). Only mice with port-site tumors (*yellow broken circles*) were used for analysis. Tumors derived from the injected CT26 cells were confirmed by Cherry-Red fluorescence (Supplementary Fig. 2). Number of tumors counted was significantly higher in the dry-cold cohort compared to humidified-warm cohort (*p* = 0.03)

Maintaining body temperatures during laparoscopic insufflation is a challenge in patients.^22^ Similarly in our mouse model achieving temperatures between 36.5 and 37.5 °C using dry-cold CO_2_ for insufflation was difficult requiring continuous intervention with a heat lamp. At 10 min, 10 of 14 and 4 of 14; at 20 min, 8 of 14 and 1 of 14; and at 30 min, 7 of 14 and 1 of 14 mice in dry-cold and humidified-warm cohorts, respectively, required significantly more heating intervention due to body temperatures falling below 36.5 °C. During the first 30 min, and despite persistent external heating, the rectal (core body) temperature was significantly lower (*p* < 0.02) in mice insufflated with dry-cold CO_2_, with normalization not achieved until 40 min (Fig. 2a).

### Humidified-Warm CO_2_ Reduces Peritoneal Metastasis

Mice were collected 10 days after tumor cell inoculation and peritoneal tumor formation assessed (Fig. 2b). A minimal tumor cell inoculum of 1000 cells was used to explore the establishment of tumors from the lowest practical cell number while maintaining consistent tumor development at inherently damaged port sites. The presence of port-site tumors further served as a methodology control confirming tumor cell viability and invasive potential.

All mice developed at least one of two port-site (inlet or exit port) tumors, presumably due to tissue damage caused by the catheters. Port-site tumors were excluded from the analysis of peritoneal tumor burden in all cohorts of mice because these were not attributable to mesothelial damage cause by CO_2_ insufflation per se. Of the dry-cold and humidified-warm cohorts, 8 of 14 and 4 of 14 mice developed peritoneal tumors, respectively (Fig. 2b). Tumors were confirmed and assessed using fluorescence imaging of Cherry-Red-tagged tumor cells (Supplementary Fig. 2). A significantly higher (*p* < 0.03) tumor burden was found in mice exposed to the dry-cold CO_2_ compared to humidified-warm CO_2_. Ten of fourteen (71 %) mice in the humidified-warm cohort did not have any tumors with detectable fluorescent expression, while in the dry-cold insufflation cohort, 6 of 14 mice (43 %) remained free of tumor.

### Mesothelial Surface Damage after Insufflation

**Fig. 3:**
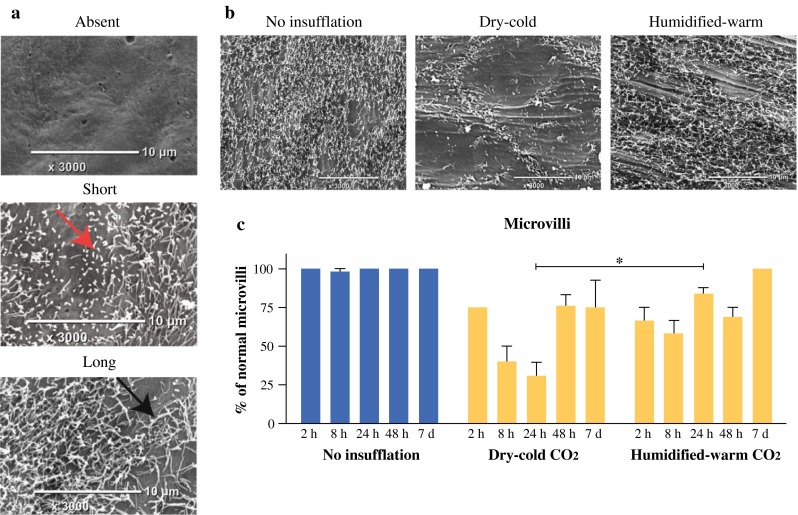
Mesothelial microvilli integrity is disrupted after CO_2_ insufflation. **a** Regions where microvilli were essentially absent or were shorter than normal. *Red arrow* indicates typical example of shortened microvilli; *black arrow,* long microvilli. **b** Representative SEM images of the peritoneum at 24 h for each treatment group. **c** Quantitation of microvilli status in each cohort. Comparative statistical analyses between groups are tabulated (*p < 0.001; *t* test)

SEM images of the apical mesothelial surface without exposure to insufflation demonstrate extensive and uniform microvilli. Insufflation led to significant aberrations to microvilli, including reduced density and shortening (Fig. 3a, b). Dry-cold CO_2_ exposure was consistently associated with shortened microvilli and regions where villi were absent. At 24 h, microvilli damage was more severe in the dry-cold exposed samples compared to the other groups (*p* < 0.001) (Fig. 3c).

### Mesothelial Alterations and Tumor Cell Adhesion

**Fig. 4:**
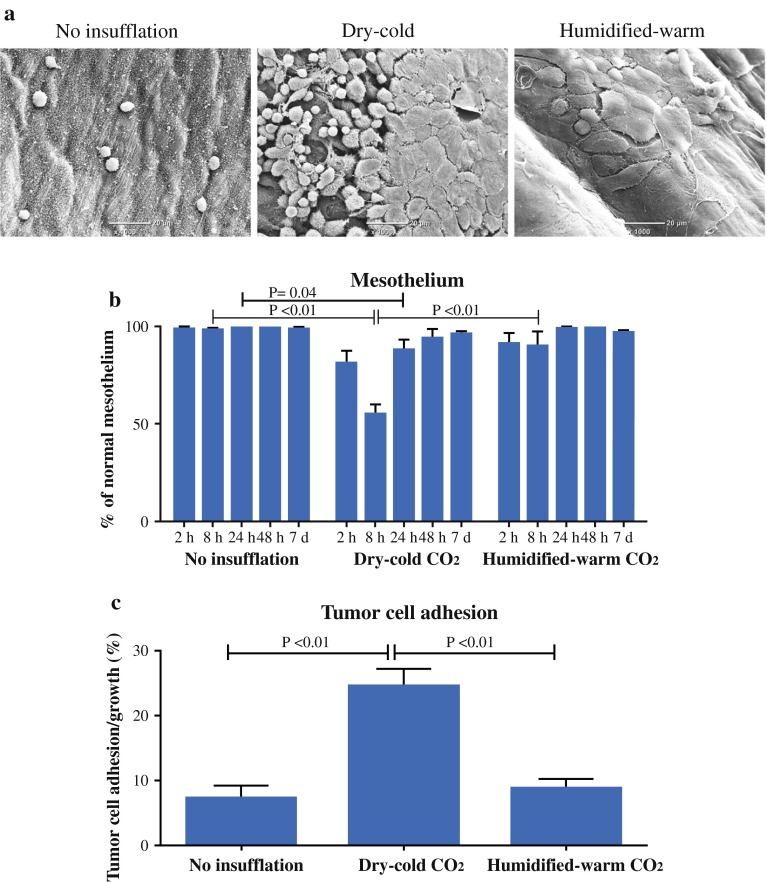
Mesothelial layer integrity and cancer cell adhesion after CO_2_ insufflation. **a** Representative SEM images of peritoneum captured for each treatment group at 8 h. **b** Quantitation of rounding up and retracting cells for different time points. **c** Tumor cell adhesion quantified for each treatment group showing elevated adhesion in the dry-cold CO_2_ group (*t* test)

To assess the relationship between prolonged insufflation, mesothelial alterations, and tumor cell adhesion, the presence of rounding and retraction of cells away from each other were used as measures of early delamination of the mesothelium from the basement membrane and cellular damage (Fig. 4a). There were significantly more rounded mesothelial cells in dry-cold CO_2_ compared to humidified-warm CO_2_ samples at 8 h (Fig. 4b).

To allow identification of tumor cell attachment to damaged peritoneum, 10^6^ cells were delivered; then the peritoneum was evaluated by SEM and for Cherry-Red expression by IHC (Supplementary Fig. 3). Collectively, more single and diffuse tumor clusters adhered to the peritoneum under dry-cold compared to humidified-warm CO_2_ samples (Fig. 4c).

### Mesothelial Inflammation After Insufflation

**Fig. 5:**
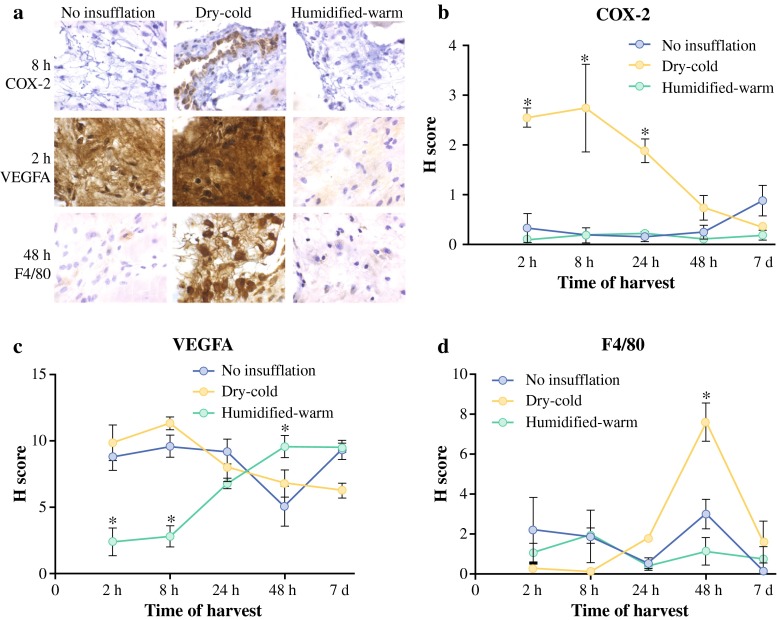
Markers of inflammation and macrophage infiltration change after CO_2_ insufflation. **a** Visualization of markers and semiquantitative analyses were performed by scoring extent by intensity of antigen expression (*brown staining*) averaged per mouse at five different areas of mesothelium in three mice per treatment per time point (original magnification, ×40). **b** COX-2. **c** VEGFA. **d** F4/80 macrophage marker (*p < 0.05; *t* test)

The effect of different CO_2_ conditions on the production of inflammatory mediators was assessed in peritoneal tissue at different times after insufflation by IHC (Fig. 5a). Inflammatory marker COX-2 was significantly increased (*p* < 0.01) until 48 h after insufflation in the mesothelium of mice insufflated with dry-cold CO_2_. In contrast, COX-2 expression within the mesothelium was indistinguishable in the humidified-warm CO_2_ and noninsufflated controls (Fig. 5b). By 7 days, COX-2 expression was not significantly different.

VEGFA, a marker of inflammation and angiogenesis, was normally expressed on the mesothelium. VEGFA levels were significantly reduced in tissue exposed to humidified-warm CO_2_ in the first 8 h (*p* < 0.01) but returned to levels comparable to the control by 24 h after the procedure (Fig. 5c).

Finally, a central response to tissue damage is the infiltration of macrophages that are charged with the task of removing cell debris and optimizing tissue regeneration.^23^,^24^ Accordingly, macrophage infiltration was assessed using the characteristic marker F4/80. Macrophage levels were similar up to 24 h, but by 48 h, macrophage infiltration was significantly increased (*p* < 0.01) in mice exposed to dry-cold CO_2_ ( Fig. 5d).

## Discussion

Laparoscopy is commonly used for many conditions, including cancer resection, and potentially when unrecognized cancer may be present within the abdominal cavity.^25^ Here we have modeled aspects of laparoscopy in mice focusing on CO_2_ gas conditions for insufflation. The rationale for using humidified-warm CO_2_ gas over readily available dry-cold CO_2_ for insufflation has previously concentrated on hypothermia and tissue plane adhesions.^2^,^26^,^27^ The novelty of this study is that we developed and optimized a mouse model of laparoscopic insufflation to investigate the cellular consequences of standard dry-cold CO_2_ on inflammation and tumor adhesion. The most immediate observation was that core-body temperature was more readily maintained with humidified recapitulated observations previously reported but also documented and quantified for the first time differences at the subcellular level.^5^

Peritoneal mesothelial cells are carpeted with microvilli where they serve to increase the surface area and perhaps augment a physical barrier to damage. Significantly reduced microvilli densities and marked villus shortening were found with dry-cold at 24 h compared to the humidified-warm CO_2_ cohort. Significant mesothelial cell perturbations characterized by rounding and retraction were also observed after dry-cold CO_2_ insufflation.

Tumor cell adhesion to the peritoneum was increased commensurate with the elevated damage caused by dry-cold CO_2_ after insufflation and cancer cell injection. Significantly less tumor cell adhesion was evident after humidified-warm CO_2_, suggesting enhanced protection and immune clearance of cancer cells. Consistent with enhanced mesothelial damage, when assessing inflammation in each CO_2_ group, elevated and prolonged COX-2 expression was measured in the dry-cold cohort. The basis for this is unclear, but a reasonable possibility is that the tissue damage invokes this inflammatory response. By 7 days in all sections, the acute inflammatory response to the dry-cold insufflation was resolved.

We identified an unexpected reduction in VEGFA expression by humidified-warm CO_2_. This suggests that humidified-warm CO_2_ actively reduces VEGFA below normal homeostatic levels. Such a suggestion is consistent with the data from the no-insufflation controls, which exhibited minimal mesothelial damage but also had the same apparent level of VEGFA.

An intriguing possible reason for the effects on VEGFA expression is that it is induced by the presence of the tumor cells, consistent with other reports, and that humidified-warm CO_2_ actively blocks VEGFA expression.^22^ This might suggest an unexpected benefit from using this gas condition for insufflation. The reduction in VEGFA is also likely to reduce angiogenic responses exploited by tumor cells.

In the no-insufflation and humidified-warm cohorts, delayed infiltration of macrophages was measured compared to a significant increase at 48 h in the dry-cold cohort. These data are consistent with the hypothesis that the elevated morphologic damage and increased inflammatory response stimulate increased migration of macrophages to sites of damage for the purpose of aiding in tissue repair.^23^

The early embedding of tumor cells to insufflation-mediated damaged peritoneum was significantly greater in the dry-cold insufflated cohort of mice in which a larger peritoneal tumor burden was identified compared to humidified-warm gas insufflation. However, established tumor growth rate was not influenced by either gas, suggesting peritoneal damage influenced tumor cell adhesion but not the rate of subsequent tumor growth. Consistent with this view was that cancer cell adhesion to port sites was a general feature of the model we used here, as well as the overall increased propensity for cancer cell adhesion to areas of damaged tissue. These data highlight to the importance of minimizing tissue damage and consequent peritoneal inflammation even when the tumor burden is relatively minimal.

Our study has limitations, as it is based on studies in mice. Although the mechanisms underpinning COX-2 expression are well understood^22^, the basis for our observation that VEGFA suppression in the context of tumor cells in the peritoneum is unclear and requires future investigation. Similarly, there is an imperative that the expression of these proteins in human peritoneum will need to be established under different insufflation conditions before our mouse studies can be credibly translated to humans. An additional limitation is the surgical intervention within the peritoneum at the time of insufflation compared to patients undergoing surgery. Because the difference in peritoneal damage and implantation between the two CO_2_ modalities is so significant in the mouse model, it is likely that this will only be exacerbated by the addition of mechanical trauma. Nevertheless, the long-term benefits of laparoscopically assisted surgery in the management of colorectal cancer over open surgery remains, so interventions that minimize adverse events associated with laparoscopy are of potential merit.^24^ One potential intervention within convenient reach is to consider the use of COX-2 inhibitors, either contemporaneously or after insufflation and/or surgery to reduce peritoneal inflammation, particularly when dry-cold CO_2_ is used. The consequence of this intervention could be addressed experimentally in laparoscopic models like that reported here in the future.

In conclusion, by using humidified-warm CO_2_ for laparoscopic insufflation in a mouse model, we demonstrated that core temperature is more easily maintained during surgery, peritoneal damage and inflammation are reduced, and, most importantly, the subsequent development of peritoneal metastasis after surgery is lowered compared to conventional dry-cold CO_2_ insufflation. Our observations also suggest that the expression of tumor-promoting mediators is exacerbated by using dry-cold CO_2_.

## Electronic supplementary material

Below is the link to the electronic supplementary material.

Supplementary material 1 (DOCX 25 kb)

Supplementary material 1 (TIF 2518 kb)

Supplementary material 1 (TIF 1183 kb)

Supplementary material 1 (TIF 4262 kb)
