# Supplementary material for: Peritoneal Tumorigenesis and Inflammation are Ameliorated by Humidified-Warm Carbon Dioxide Insufflation in the Mouse
Source: Ann Surg Oncol. 2015 Mar 21;22(Suppl 3):1540–7. doi: 10.1245/s10434-015-4508-1 (PMC4687477; doi:10.1245/s10434-015-4508-1)
Supplement: Supplementary file 1 — Supplementary material 1 (DOCX 25 kb) [file 10434_2015_4508_MOESM1_ESM.docx]

**Supplementary Information**

***Detailed Insufflation Procedures and Experimental Design***

To model the consequences of potential tumor cell mobilization and engraftment following laparoscopic insufflation conditions, BALB/c mice were anaesthetized using isofluorane inhalant (Abbott Laboratories, Pty Ltd, Australia), intubated with a 20G catheter, connected to a ventilator (MiniVent Harvard Apparatus, MA, USA) set to 200 strokes/min, 200 µl stroke volume and maintained under anaesthesia with 2.5 - 5.0% isofluorane. IP- anesthetic delivery such as Pentobarbital was avoided as these can cause peritoneal irritation. Ventilation was provided to avoid a hypoxic peritoneal environment. Two 18G catheters were introduced intra-peritoneally. The ‘exit port’ catheter was connected to a valve device whereas the ‘entry port’ catheter was connected to tubing that delivered CO_2_ into the abdominal cavity. Mice were insufflated for 1 h with either dry-cold or humidified-warm CO_2._ The physical set-up and intervention points/port-sites are described in Supplementary Fig. 1 and Fig. 1a & c.

Following one hour of insufflation, connections to port catheters were detached to allow de-sufflation. A density of 1 x 10^3^ or 1 x 10^6^ murine colorectal cancer cells (CT26[^21^](#_ENREF_21) - tagged with Cherry-Red) in 200 µl sterile phosphate buffered saline (PBS) were injected through the entry port into the peritoneal cavity (Supplementary Fig. 1). The port catheters were removed, Rimadyl analgesic at 4 mg/kg was delivered sub-cutaneously (Carprofen, 50 mg/ml, Pfizer, supplied by Ellar) and the mouse was removed from the ventilator to allow recovery from anaesthesia. Control mice were connected to a ventilator, maintained under anesthesia for 1 h, port-sites were introduced into the peritoneum but these were plugged to prevent any external gas exchange. CT26 cells were then delivered.

For humidified-warm CO_2_ insufflation, the upper chamber of the HumiGard™ (Fisher & Paykel Healthcare, NZ) was filled with sterile distilled water, pre-warmed and maintained at 37 ^o^C with 100% relative humidity. For dry-cold CO_2_ delivery, the device was not filled with water and the heater was turned off. Flow rates and pressure was maintained within the range of 14-52 ml/min at 2 mmHg pressure. The pressure and flow rates were calculated based on mouse size and were relative to human laparoscopy following observations in pilot trials where flow rates above 200 ml/min resulted in peritoneal damage only in the path of flow (Fig. 1b). Mice were kept on a heating pad and a rectal probe (Able scientific) was used to monitor temperature. If temperature fell below 36.5 ^o^C, an infrared heating lamp (Nesco) was used.

To analyse peritoneal tumor formation following different CO_2_ conditions, mice were harvested 10 days following tumor cell injection and counted macroscopically. Ten days allowed peritoneal tumor development that would be detected by imaging software. CT26 CherryRed cells definitively allowed identification of tumors and these were quantified using fluorescence imaging. The peritoneum was harvested at different time points for immunohistochemistry (IHC) and scanning electron microscopy (SEM) analyses. The experimental design is detailed and illustrated in Fig. 1c. *End-point experiments:* No insufflation, n=3; Dry-cold CO_2_ insufflation, n=14; Humidified-warm CO_2_ insufflation, n=14. *Time course experiments:* No insufflation, n=15; Dry-cold CO_2_ insufflation, n=15; Humidified-warm CO_2_ insufflation, n=15. Within each group at each time point 2, 8, 24, 48 h and 7 days, n=3.

***Immunohistochemistry (IHC)***

Sections were de-waxed and rehydrated. Antigen retrieval was performed in a pressure cooker as outlined below and endogenous peroxidase blocked with 3% H_2_O_2_ for 10 min. Sections were washed with Tris-Tween 0.1% pH 7.6 buffer then incubated with appropriate primary antibody in a humidified chamber. Sections were washed 3 times, then incubated with appropriate secondary antibody for 30 min, washed again 3 times and then developed with DAB+chromagen. Slides counterstained with haematoxylin and mounted in Entellan Medium.

Antibody dilutions were used as follows: COX-2 (sc-1745, Santa Cruz), 1:1000 at 4^o^C overnight. Antigen retrieval was performed with 1mM EDTA pH 8. VEGFA (DP3520S, Acris) 1:100 at 4^o^C overnight. Antigen retrieval was performed with Trypsin (Calbiochem). F4/80 (123101, Biolegend), 1:500 for 1 h at RT^o^C. Antigen retrieval was performed with 1 X proteinase K (Abcam). mCherry (ab125096, Abcam), 1:1000 for 1 h at RT^o^C. Antigen retrieval was performed with 1 mM EDTA pH 8.

***Tissue Harvesting and Preparation***

At the time of harvest the mice were culled by cervical dislocation. The peritoneum was isolated and pinned to a Balsa wood platform for tumor evaluation and fluorescent imaging or isolated, placed on a sheet of dental wax and cut in half with a double edged stainless steel blade. Half of the tissue was pinned to Balsa wood to, submerged in 10% Neutral Buffered Formalin to be fixed flat overnight, then dehydrated, paraffin embedded and sectioned horizontally (3µm) for IHC. The other half was fixed (2.5% glutaraldehyde, 2% paraformaldehyde in 0.8M Sorensen’s phosphate). further divided into a grid of six pieces and individually stored in fixative for 2 h until washing (0.08M Sorensen’s phosphate buffer) and storage in buffer (0.08M Sorensen’s phosphate buffer with 5% sucrose). Samples were dehydrated through alcohol, critical point dried using liquid CO_2_ as the exchange medium (Leica EM CPD300), mounted onto aluminium stubs and sputter coated with gold for SEM analysis.

**Supplementary Figure Legends**

**Supplementary Fig 1.** The set-up for ventilation with anesthetic/oxygen delivery in mice is shown highlighting precise locations of ports (green circles) .

**Supplementary Fig 2.** There was no significant difference between the average fluorescence signal of individual peritoneal tumors in the dry-cold compared to humidified-warm insufflated mice (p=0.16), (t-test). Tumors visualized macroscopically in **Fig 2** are confirmed as being derived from Cherry Red-labelled CT26 colorectal cancer cells.

**Supplementary Fig. 3** Mesothelial layer integrity and cancer cell adhesion after CO_2_ insufflation. **a** An example of a region where mesothelial cells were rounded (Ro) or retracted (Re) exposing the basal lamina is depicted. **b** The presence of attached CT26 tumor cells was determined by morphological criteria using SEM as well as by Cherry Red IHC (brown-stained cells) prepared from the same peritoneum indicated by arrows (Magnification 40X).
