# Supplementary figures and images for: Peritoneal Tumorigenesis and Inflammation are Ameliorated by Humidified-Warm Carbon Dioxide Insufflation in the Mouse
Source: Ann Surg Oncol. 2015 Mar 21;22(Suppl 3):1540–7. doi: 10.1245/s10434-015-4508-1 (PMC4687477; doi:10.1245/s10434-015-4508-1)

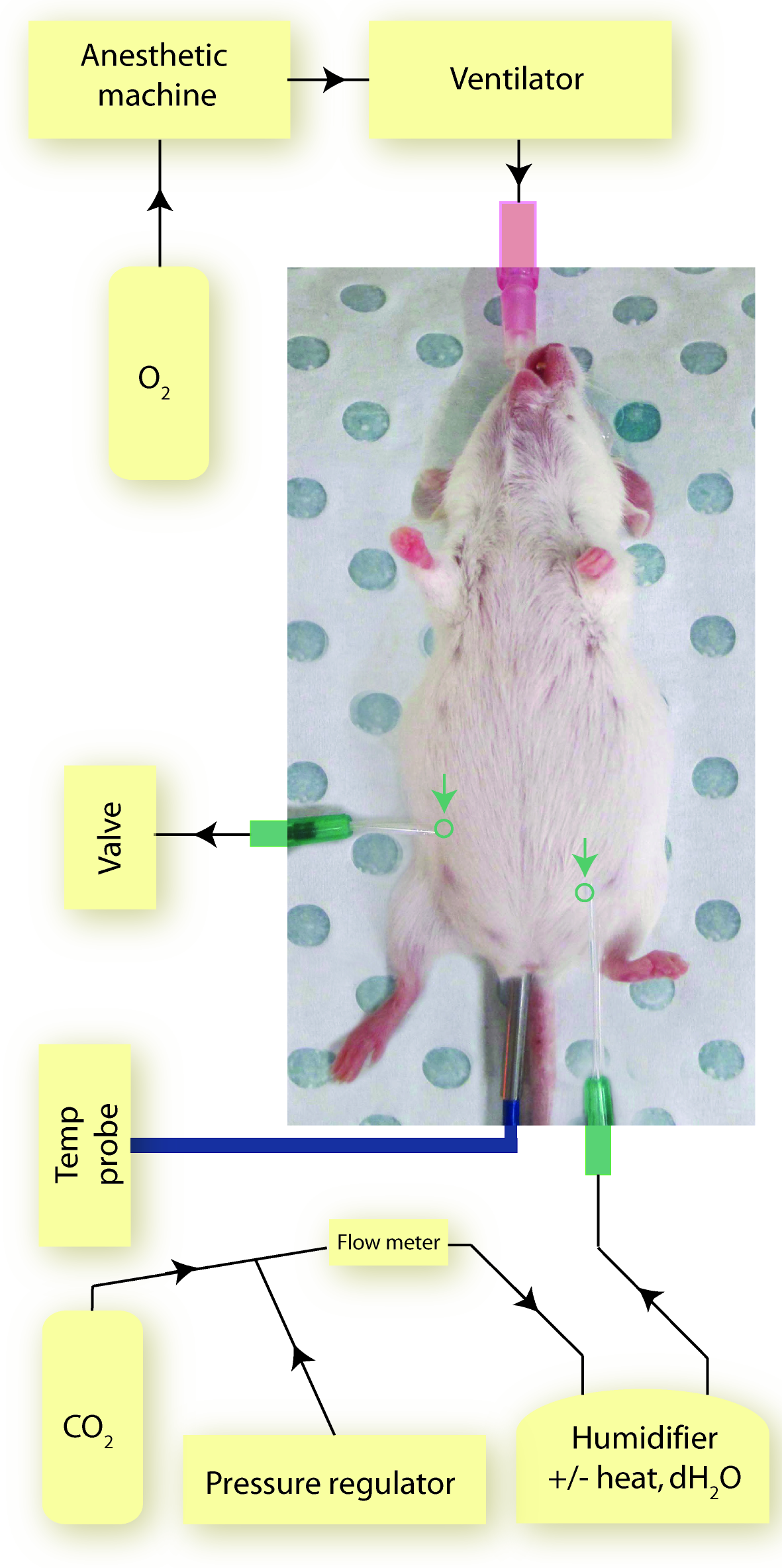

Supplement: Supplementary file 2 — Supplementary material 1 (TIF 2518 kb) [file 10434_2015_4508_MOESM2_ESM.tif]

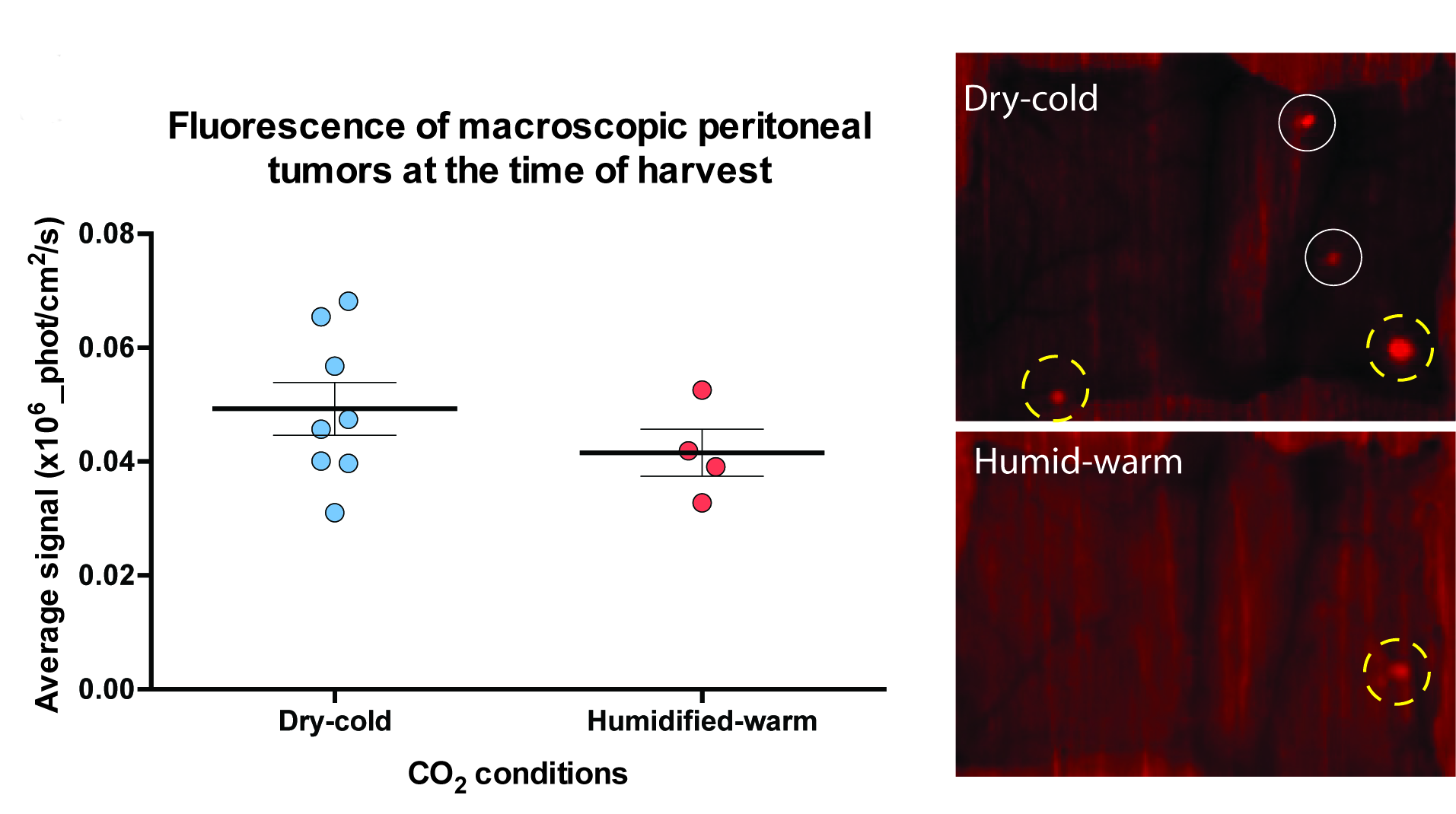

Supplement: Supplementary file 3 — Supplementary material 1 (TIF 1183 kb) [file 10434_2015_4508_MOESM3_ESM.tif]

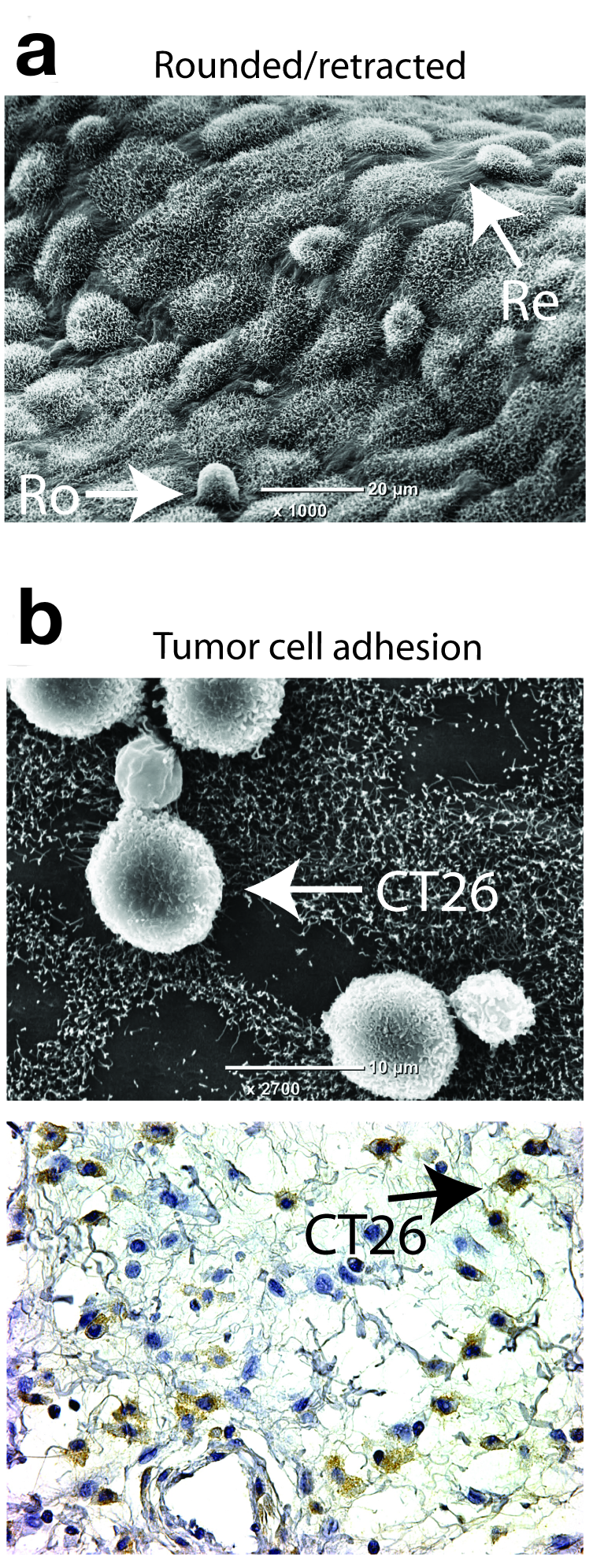

Supplement: Supplementary file 4 — Supplementary material 1 (TIF 4262 kb) [file 10434_2015_4508_MOESM4_ESM.tif]
